# Involuntary psychiatric hospitalisation – differences and similarities between patients detained under the mental health act and according to the legal guardianship legislation

**DOI:** 10.1186/s12888-024-05892-z

**Published:** 2024-06-13

**Authors:** Sönke Johann Peters, Mario Schmitz-Buhl, Jürgen Zielasek, Euphrosyne Gouzoulis-Mayfrank

**Affiliations:** 1 LVR Institute for Healthcare Research, Wilhelm-Griesinger-Strasse 23, 51109 Cologne, Germany; 2 LVR Clinics Cologne, Wilhelm-Griesinger-Strasse 23, 51109 Cologne, Germany; 3 Medical Faculty, Heinrich Heine University Düsseldorf, Universitätsstraße 1, 40225 Düsseldorf, Germany

**Keywords:** Mental Health Act, Legal guardianship, Involuntary hospitalisation, Coercion, Machine learning, Random Forest

## Abstract

**Background:**

Involuntary psychiatric hospitalisation occurs under different legal premises. According to German law, detention under the Mental Health Act (MHA) is possible in cases of imminent danger of self-harm or harm to others, while detention according to the legal guardianship legislation (LGL) serves to prevent self-harm if there is considerable but not necessarily imminent danger. This study aims to compare clinical, sociodemographic and environmental socioeconomic differences and similarities between patients hospitalised under either the MHA or LGL.

**Methods:**

We conducted a retrospective health records analysis of all involuntarily hospitalised cases in the four psychiatric hospitals of the city of Cologne, Germany, in 2011. Of the 1,773 cases, 87.3% were detained under the MHA of the federal state of North Rhine-Westphalia and 6.4% were hospitalised according to the federal LGL. Another 6.3% of the cases were originally admitted under the MHA, but the legal basis of detention was converted to LGL during the inpatient psychiatric stay (MHA→LGL cases). We compared sociodemographic, clinical, systemic and environmental socioeconomic (ESED) variables of the three groups by means of descriptive statistics. We also trained and tested a machine learning-based algorithm to predict class membership of the involuntary modes of psychiatric inpatient care.

**Results:**

Cases with an admission under the premises of LGL lived less often on their own, and they were more often retired compared to MHA cases. They more often had received previous outpatient or inpatient treatment than MHA cases, they were more often diagnosed with a psychotic disorder and they lived in neighbourhoods that were on average more socially advantaged. MHA→LGL cases were on average older and more often retired than MHA cases. More often, they had a main diagnosis of an organic mental disorder compared to both MHA and LGL cases. Also, they less often received previous psychiatric inpatient treatment compared to LGL cases. The reason for detention (self-harm or harm to others) did not differ between the three groups. The proportion of LGL and MHA cases differed between the four hospitals. Effect sizes were mostly small and the balanced accuracy of the Random Forest was low.

**Conclusion:**

We found some plausible differences in patient characteristics depending on the legal foundation of the involuntary psychiatric hospitalisation. The differences relate to clinical, sociodemographic and socioeconomical issues. However, the low effect sizes and the limited accuracy of the machine learning models indicate that the investigated variables do not sufficiently explain the respective choice of the legal framework. In addition, we found some indication for possibly different interpretation and handling of the premises of the law in practice. Our findings pose the need for further research in this field.

## Background

Laws allowing involuntary admission to psychiatric hospitals in case of risk of self-harm or harm to others are common legal instruments and exist in all European countries and many other countries [1–3]. Common legal options for involuntary hospitalisation are the Mental Health Act (MHA) and legal guardianship legislation (LGL). However, the prerequisites for the application of the laws differ from country to country.

In Germany, patients are eligible for involuntary hospitalisation based on the MHA of the federal states in case of imminent risk of self-harm or harm to others, while involuntary hospitalisation under LGL is only admissible for the welfare of the person under guardianship. The threat of self-harm needs to be considerable in order to justify detention under LGL, but it may not be necessarily imminent. Thus, LGL in contrast to MHA considers behaviour that may cause self-harm in the future. The length of the involuntary hospitalisation varies broadly under both legal premises. However, for an involuntary hospitalisation under the MHA ordered by injunction if exceeding six weeks, usually a new court hearing will be held. If the overall duration exceeds three months, an injunction by the court is not sufficient and an additional psychiatric report by an external expert is commissioned [4–6]. Commonly, involuntary hospitalisations under LGL have a longer duration right from the beginning because court orders by means of an injunction are not common. The total duration of involuntary hospitalisation ranges from a few weeks up to a year or more.

The MHA allows involuntary medication only for the management of the respective psychiatric disorder. Under German LGL on the other hand it is also possible to diagnose and treat non-psychiatric conditions under coercion if otherwise a considerable damage to the patient’s health is expected. Any treatment or diagnostic procedure under coercion upon request of the legal guardian nevertheless needs to be approved by the guardianship court [7].

Evidence on detention and coercive measures according to LGL in European countries is scarce. After the United Nations ratified the Convention on the Rights of Persons with Disabilities in 2008, the European Court of Human Rights found for example that the Bulgarian and Czech guardianship laws violated the convention, because a person with schizophrenia was denied to challenge their confinement [8]. However, we are not aware of any international publication describing changes in the LGL in the respective countries over time. Most publications focus on MHA legislation. In Finland, for example, there seems to be no legislative distinction between involuntary hospitalisation and involuntary treatment under the MHA. The latter is only applicable to patients with a psychotic disorder including delirium and severe dementia [9]. More evidence exists on the use of LGL in non-European countries: In Australia, the MHA is commonly used for psychiatric treatment in case of self-harm and harm to others. If other medical treatment is needed as well, the Guardianship Act can be used instead [10]. In Japan, legal guardianship is used for involuntary hospitalisation of minors *and* adults while in Thailand, guardianship laws apply only to minors [11]. Finally, in the USA, involuntary psychiatric admission criteria vary from state to state [12] and involuntary admission of elderly people with organic mental disorders such as Alzheimer’s disease is inconsistently managed: Many medical directors of psychiatric hospitals in the USA see the use of durable power of attorney for health care as sufficient for involuntary hospitalisation of this group, while others advocate the use of standard involuntary commitment procedures [13].

Given these considerable differences of the legal pathways to involuntary psychiatric hospitalisation, one would expect that patient characteristics differ between MHA and LGL groups of patients. Identifying and analysing such differences may be helpful to elucidate more specific ways of preventing involuntary hospitalisation for these disparate patient groups. Both clinical and social factors may play a role. Previous studies summarised in a recent meta-analysis of clinical and social factors associated with an increased risk for involuntary hospitalisation [14] mostly focused on involuntary psychiatric hospitalisation under the respective Mental Health Act (MHA). Overall, we found little evidence on the subject of involuntary psychiatric hospitalisation and treatment under legal guardianship legislation (LGL). Some research groups explicitly exclude guardianship cases from analyses on risk factors for involuntary admission [15]. Overall, the insight from studies into legal conditions and practical application of involuntary admission under LGL as well as characteristics of people who are detained in a psychiatric hospital according to LGL is very limited. Furthermore, many studies focus on patients with dementia and do not address other mental health conditions that may cause the appointment of a legal guardian [16].

Thus, it is still unclear whether risk factors for involuntary hospitalisation differ between detained MHA and LGL patients. Differences between these two subgroups could be relevant to better understand the factors contributing to involuntary hospitalisation and to develop preventive measures specifically targeted to the MHA and LGL groups, respectively. Therefore, we analysed all involuntarily hospitalised cases of a year in a metropolitan city of Germany in regard to sociodemographic, clinical, systemic and socioeconomic variables.

## Methods

### Setting

The clinical routine dataset used in this study contains the records of all adults with mental disorders who were detained under the MHA or according to the LGL in mental health hospitals in the city of Cologne from January 1st to December 31st in 2011. Cologne is Germany’s fourth largest city with about one million inhabitants. In 2011, psychiatric inpatient treatment in Cologne was provided by four hospitals. Each of the hospitals provides statutory psychiatric emergency services in defined geographical sectors of the city with approximately 100,000 to 500,000 inhabitants each. The LGL is specified in the German Civil Code (Bürgerliches Gesetzbuch – BGB) which is federal law, while the MHA falls under the federal state legislation. Therefore, federal BGB law applies to all LGL cases and the Mental Health Act of North Rhine Westphalia (Gesetz über Hilfen und Schutzmaßnahmen bei psychischen Krankheiten – PsychKG NRW) applies to all MHA cases.

Involuntary hospitalisation under the PsychKG NRW needs to be initiated by “a physician with experience in the field of psychiatry”. On the same day or on the day after admission, a court hearing by the local court needs to be held to confirm or revoke the detention. Involuntary admission under LGL is initiated by the patient’s guardian and needs to be authorised by the local court in advance or shortly after confinement on the same day. The same local court was responsible for all involuntary hospitalisations in Cologne in 2011.

### Data sources and study design

The data used for this retrospective study are part of a bigger dataset used in previous studies [17–19]. The dataset contains administrative, clinical, sociodemographic and socioeconomic data that were extracted by five assistant physicians from the patient health records of the patients. The dataset included both the main diagnosis, which was the reason and main target of the inpatient treatment, and all secondary diagnoses, i.e. psychiatric comorbidities. All diagnoses were made and recorded according to WHO ICD-10 classification [20]. The dataset also included the gender, age, information on marital/ relationship status and children, migration background, housing situation, education and professional situation. It furthermore contained information about the psychiatric treatment history, the length of the current inpatient stay, and information on suicidal tendencies. The dataset was enriched by environmental socioeconomic data (ESED) characterising the living environment of the patients. The ESED were obtained from RWI-GEO-GRID [21] providing information on environmental socioeconomic key indicators based on 1 × 1 km small grid cells linked to the postal code of the patients’ home address [22]. All variables used in this study are shown in Table 1. For more background information on the collection of the data, we refer to our previous publications [17–19].

**Table 1:** Variables included in the analysis

Category	Levels
**Sociodemographic characteristics**	
Gender	Female, male
Age	
Age (by age group)	≤ 40, 41–60, > 60 years of age
Marital status	Single, married, widowed, divorced, living apart
Relationship	Yes, no
Children	Yes, no
Migration background	Yes, no
Living situation	Alone, family/ partner, community, assisted accommodation, emergency accommodation/ homeless
School education	No graduation, lower secondary school, higher secondary school, A-levels
Professional education	None, apprenticeship, master apprenticeship, university
Professional situation	Employed, unemployed, homemaker, retired, in training
Degree of employment	None, full time, part time
Main source of income	Employment, pension, own assets, unemployment benefits, alimony
**Clinical and systemic characteristics**	
Main diagnosis (ICD-10)	F0, F1, F2, F3, F4, F6, F7, F9, other
Main or secondary diagnoses (ICD-10)	F0, F1, F2, F3, F4, F6, F7, F9
Dual diagnoses (comorbidities)	F1 + F2, F1 + F6
Suicidal tendencies upon admission	Yes, no
Previously attempted suicides	Yes, no
Reason for involuntary treatment	Danger of self-harm, danger of harm to others, both
Previous psychiatric outpatient treatment	Yes, no
Previous psychiatric inpatient treatment	Yes, no
Treating hospital	Hospital 1, hospital 2, hospital 3, hospital 4
Time of admission	Regular service hours, outside service hours
Length of inpatient stay	Number of days
**Environmental socioeconomic characteristics**	
Number of commercial enterprises per 100 inhabitants	
Unemployment rate per 100 inhabitants	
Employment rate per 100 inhabitants	
Number of buildings per 100 inhabitants	
Number of residential buildings per 100 inhabitants	
Number of households per 100 inhabitants	
Number of children per 100 inhabitants	
Purchasing power per 100 inhabitants [€]	

### Patient population

Data were anonymised and one patient may cause more than one case if they have been admitted more than once in 2011. Therefore, we use the term *cases* instead of *patients* in this study We compared 1,548 cases who were treated under the MHA with 114 cases treated under LGL and another 111 cases who were detained under the MHA at admission, but later during treatment, the legal basis for involuntary treatment was converted to LGL (MHA→LGL cases). There were no cases admitted under LGL who later changed to MHA. In a previous publication, we had investigated differences between cases hospitalised under the MHA since admission and those detained later in the course of treatment. These groups were largely similar regarding clinical and sociodemographic characteristics [19]. Therefore, in line with our previous findings, we did not differentiate between these subgroups. However, we did analyse the MHA→LGL cases separately because the reasons for the conversion of the legal status may lie in specific features of the patients. For example, some patients with an acute exacerbation of a psychotic disorder may be initially admitted due to aggressiveness and danger of harm to others under the premises of MHA. In the course of inpatient treatment and medication, aggressiveness often attenuates and more long-term related dangers like self-neglect may become dominant and require continuation of the involuntary hospitalisation under the premises of LGL. In other cases, during an involuntary psychiatric inpatient stay under the MHA, a legal guardian may get newly appointed because the need for long-term assistance becomes apparent. For these reasons, we treated these cases in the descriptive and inferential statistics analysis as a separate group. However, as the primary research question was to discover potential differences between MHA and LGL cases, we excluded the MHA→LGL cases from the machine learning analysis.

### Statistical analysis

For the descriptive statistics, we compared the three groups of cases under the MHA, under LGL, and with conversion from MHA to LGL during the inpatient stay. We used Chi-squared test for the categorical data like diagnoses, sociodemographic data and other clinical details. We analysed both main and secondary diagnoses of mental disorders. Additionally, we analysed the comorbid occurrence of addiction and psychosis (F1 and F2), and addiction and personality disorder (F1 and F6), as these comorbidities are linked to higher rates of self-harm, aggressive behaviour, impulsivity and violent crimes [23–26]. For the metric data like age, length of inpatient stay and the ESED, we used analysis of variance (ANOVA). We used Holm corrections for the pairwise comparison of the Chi-squared tests to differentiate between the three investigated subgroups. For the ANOVA, we used Tukey’s honest significant differences test (Tukey-HSD). Due to the high number of variables overall investigated, we chose a conservative level of significance of *p* ≤ 0.01. Because of the already conservative level of significance and the exploratory nature of the study, we refrained from additional corrections for multiple testing [27]. We used eta-squared for ANOVA tests and Cramér’s V s for Chi-squared tests to calculate effect sizes for the variables with a significant result. For the purpose of better readability, we only report the test details of variables with significant differences.

In order to identify variables for the prediction of the legal basis of a detention, we used a Random Forest machine learning algorithm. The Random Forest algorithm selects random subsets and random variables of the dataset and creates multiple decision trees. This process called bootstrapping helps avoiding overfitting, a common problem with decision tree algorithms, and it is relatively robust to outliers and noise [28]. Tree-based algorithms are particularly beneficial when the objective is not to test for the association of a single variable or small set of variables, but rather for describing associations in bigger datasets. In comparison to other tree-based prediction algorithms like Chi-square-Automation-Interaction-Detection (CHAID) and Classification and Regression Tree analysis (CART), the Random Forest algorithm usually yields a higher prediction accuracy and more stable prediction models. The higher accuracy is achieved because it is based not on a single, but on multiple trees created from bootstrapped subsamples of the original dataset [29]. Random Forest is therefore a commonly used machine-learning algorithm and it has also found its way into psychiatric research. It has previously been used to detect prescription patterns of antipsychotics in patients with schizophrenia [30], to classify Alzheimer’s disease using clinical routine data and cognitive measures [31] and to predict mechanical restraint of psychiatric inpatients [32].

For the Random Forest model, we included all variables except for the length of the current inpatient stay, as this variable cannot be a predictor for involuntary hospitalisation. We used a 70% sample stratified by legal status to train the algorithm and the other 30% of the cases to test the trained algorithm. We compared all variables of the stratified samples for significant differences to ensure a suitable fit of the samples. We then computed a Random Forest Model based on a *complete case dataset* (*n* = 446) after deletion of all cases with at least one missing value. Given the high number of missing values, in order to avoid distortion by listwise deletion, we computed another Random Forest model after imputation of all missing values. Imputation techniques are widely seen as less prone to distortion than listwise or pairwise deletion and regarded as a useful tool for missing data in complex epidemiologic datasets [33, 34], especially when the data is not missing completely at random (MCAR) [35]. We hypothesised that in groups of the observed data, the probability of data being missing is likely to be elevated and therefore not MCAR but rather missing at random (MAR). For example, in clinical practice, a complete sociodemographic history usually exists for patients with multiple inpatient stays while it tends to be less complete in patients who are admitted for the first time and have only a short duration of inpatient stay.

For the imputation, we decided for a Random Forest-based imputation using the trees’ proximity data to replace the missing values. As the cases detained under LGL accounted for only 6.9% of the cases included in the Random Forest model, we decided to address the classification problem with another separate Random Forest model. Machine learning algorithms’ accuracy may diminish when the variable of interest in the training dataset is unequally distributed. The algorithm may be better trained on predicting the majority than the minority class, which can cause a lower balanced accuracy. We therefore used the Synthetic Minority Over-sampling Technique (SMOTE) to achieve a balanced classification in the training dataset by oversampling the minority class (LGL) with synthetic samples based on the nearest neighbours [36]. We then compared the performance of the three Random Forest models by means of confusion matrices, accuracy, sensitivity, specificity, balanced accuracy and area under the curve (AUC). For the Random Forest model with the highest balanced accuracy, we also evaluated the relevance of the variables to the model by *mean decrease in accuracy* and *Gini*, the latter being a measure of impurity of the tree node splits. All analyses were carried out with R version 4.3.0. We performed the Random Forest analysis with the randomForest package version 4.7–1.1.

## Results

All significant findings are summarised in Table 2. Details of the pairwise comparisons and effect sizes are presented in Table 3.

**Table 2:** Significant differences in the variables included in the analysis

Category	Total *N*	Missing *N*	Levels	MHA	LGL	MHA→LGL	*p*
Total *N* (%)				1548 (87.3)	114 (6.4)	111 (6.3)	
**Sociodemographic characteristics**							
Age	1773 (100.0)	0	Mean (SD)	47.4 (19.6)	46.6 (21.2)	58.8 (21.5)	< 0.001
Age (by age group)	1773 (100.0)	0	≤ 40	640 (41.3)	56 (49.1)	28 (25.2)	< 0.001
			41–60	549 (35.5)	24 (21.1)	24 (21.6)	
			> 60	359 (23.2)	34 (29.8)	59 (53.2)	
Marital status	1691 (95.4)	82	Single	786 (53.3)	73 (68.9)	56 (50.9)	0.003
			Married	315 (21.4)	11 (10.4)	15 (13.6)	
			Widowed	149 (10.1)	12 (11.3)	21 (19.1)	
			Divorced	174 (11.8)	8 (7.5)	15 (13.6)	
			Living separated	51 (3.5)	2 (1.9)	3 (2.7)	
Relationship	1519 (85.7)	254	No	546 (40.8)	26 (29.9)	19 (20.2)	< 0.001
			Yes	792 (59.2)	61 (70.1)	75 (79.8)	
Living situation	1678 (94.6)	95	Alone	563 (38.5)	24 (21.2)	41 (39.8)	< 0.001
			Family/ partner	548 (37.5)	34 (30.1)	24 (23.3)	
			Community	56 (3.8)	4 (3.5)	1 (1.0)	
			Assisted accommodation	200 (13.7)	45 (39.8)	30 (29.1)	
			Homeless	95 (6.5)	6 (5.3)	7 (6.8)	
Professional situation	1499 (84.5)	274	Employed	235 (17.9)	6 (6.5)	3 (3.1)	< 0.001
			Unemployed	489 (37.3)	32 (34.4)	23 (24.0)	
			Homemaker	77 (5.9)	3 (3.2)	4 (4.2)	
			Retired	444 (33.9)	49 (52.7)	64 (66.7)	
			In training	65 (5.0)	3 (3.2)	2 (2.1)	
Degree of employment	1453 (82.0)	320	None	1091 (86.7)	94 (97.9)	96 (97.0)	< 0.001
			Full time	132 (10.5)	0 (0.0)	1 (1.0)	
			Part time	35 (2.8)	2 (2.1)	2 (2.0)	
Main source of income	1377 (77.7)	396	Employment	231 (19.2)	5 (5.6)	2 (2.4)	< 0.001
			Pension	426 (35.4)	48 (53.3)	63 (74.1)	
			Own assets	7 (0.6)	0 (0.0)	0 (0.0)	
			Unemployment benefits	456 (37.9)	31 (34.4)	20 (23.5)	
			Alimony	82 (6.8)	6 (6.7)	0 (0.0)	
**Clinical and systemic characteristics**							
Previous psychiatric outpatient treatment	1539 (86.8)	234	No	662 (49.6)	27 (27.0)	45 (43.7)	< 0.001
			Yes	674 (50.4)	73 (73.0)	58 (56.3)	
Previous psychiatric inpatient treatment	1645 (92.8)	128	No	419 (29.4)	15 (13.2)	33 (30.8)	0.001
			Yes	1005 (70.6)	99 (86.8)	74 (69.2)	
Suicidal tendencies upon admission	1759 (99.2)	14	No	928 (60.4)	89 (78.8)	76 (69.1)	< 0.001
			Yes	608 (39.6)	24 (21.2)	34 (30.9)	
Main diagnosis	1773 (100.0)	0	F0	260 (16.8)	28 (24.6)	42 (37.8)	< 0.001
			F1	388 (25.1)	10 (8.8)	13 (11.7)	
			F2	464 (30.0)	52 (45.6)	41 (36.9)	
			F3	272 (17.6)	8 (7.0)	5 (4.5)	
			F4	83 (5.4)	3 (2.6)	2 (1.8)	
			F6	54 (3.5)	9 (7.9)	3 (2.7)	
			F7	7 (0.5)	1 (0.9)	0 (0.0)	
			F9	3 (0.2)	0 (0.0)	0 (0.0)	
			Other	17 (1.1)	3 (2.6)	5 (4.5)	
Main or secondary diagnoses: F0	1773 (100.0)	0	No	1266 (81.8)	84 (73.7)	62 (55.9)	< 0.001
			Yes	282 (18.2)	30 (26.3)	49 (44.1)	
Main or secondary diagnoses: F1	1773 (100.0)	0	No	790 (51.0)	83 (72.8)	66 (59.5)	< 0.001
			Yes	758 (49.0)	31 (27.2)	45 (40.5)	
Main or secondary diagnoses: F2	1773 (100.0)	0	No	1036 (66.9)	53 (46.5)	66 (59.5)	< 0.001
			Yes	512 (33.1)	61 (53.5)	45 (40.5)	
Main or secondary diagnoses: F3	1773 (100.0)	0	No	1176 (76.0)	98 (86.0)	101 (91.0)	< 0.001
			Yes	372 (24.0)	16 (14.0)	10 (9.0)	
Length of inpatient stay	1772 (99.9)	1	Mean (SD)	24.1 (33.2)	52.6 (51.3)	56.4 (43.8)	< 0.001
Treating hospital	1773 (100.0)	0	1	1042 (67.3)	38 (33.3)	82 (73.9)	< 0.001
			2	231 (14.9)	18 (15.8)	25 (22.5)	
			3	162 (10.5)	58 (50.9)	2 (1.8)	
			4	113 (7.3)	0 (0.0)	2 (1.8)	
**Clinical and systemic characteristics**							
Purchasing power per 100 inhabitants	1601 (90.3)	172	Mean (SD)	2189595.5 (302304.3)	2378761.8 (394662.1)	2214238.7 (338401.8)	< 0.001
Unemployment per 100 inhabitants	1601 (90.3)	172	Mean (SD)	7.5 (3.1)	6.0 (3.1)	7.3 (3.2)	< 0.001

Codes for ICD-10 diagnoses: F0: Organic mental disorders, F1: Substance use disorders, F2: Psychotic disorders, F3: Affective disorders, F4: Stress-related and somatoform disorders, F5: Behavioural syndromes associated with physical factors, F6: Personality disorders, F7: Intellectual disability, F8: Developmental disorders, F9: Behavioural and emotional disorders with onset in childhood and adolescence

**Table 3:** Pairwise comparisons and effect sizes

Category	*p*.adj MHA vs. LGL	*p*.adj MHA vs. MHA→LGL	*p*.adj LGL vs. MHA→LGL	Effect Size	
**Sociodemographic characteristics**					
Age	0.92	< 0.001	< 0.001	0.020	**
Age (by age group): ≤ 40	0.127	0.002	0.001	0.091	*
Age (by age group): 41–60	0.008	0.008	1.000	0.100	*
Age (by age group): >60	0.135	< 0.001	0.001	0.168	*
Marital status: Single	0.008	0.701	0.021	0.077	*
Marital status: Married	0.030	0.143	0.598	0.079	*
Relationship	0.115	< 0.001	0.183	0.111	*
Living situation: Alone	0.001	0.876	0.010	0.090	*
Living situation: Family/ partner	0.284	0.016	0.332	0.078	*
Living situation: Assisted accommodation	< 0.001	< 0.001	0.132	0.197	*
Professional situation: Employed	0.014	0.001	0.464	0.120	*
Professional situation: Retired	0.001	< 0.001	0.070	0.185	*
Degree of employment: None	0.007	0.010	1.000	0.113	*
Degree of employment: Full time	0.005	0.008	1.000	0.118	*
Main source of income: Employment	0.004	0.001	0.487	0.135	*
Main source of income: Retirement	0.002	< 0.001	0.007	0.206	**
**Clinical and systemic characteristics**					
Previous psychiatric outpatient treatment	< 0.001	0.186	< 0.001	0.181	*
Previous psychiatric inpatient treatment	< 0.001	0.005	< 0.001	0.310	**
Suicidal tendencies upon admission	0.016	0.089	0.466	0.088	*
Main diagnosis: F0	< 0.001	0.296	0.039	0.113	*
Main diagnosis: F1	0.001	0.841	0.005	0.093	*
Main diagnosis: F2	< 0.001	0.178	0.178	0.100	*
Main diagnosis: F3	0.090	< 0.001	0.090	0.137	*
Main diagnosis: Other	< 0.001	0.004	0.612	0.118	*
Main or secondary diagnoses: F0	0.002	0.304	0.304	0.088	*
Main or secondary diagnoses: F1	0.011	0.002	0.602	0.108	*
Main or secondary diagnoses: F2	0.631	0.028	0.690	0.075	*
Main or secondary diagnoses: F3	0.044	< 0.001	0.016	0.160	*
Length of inpatient stay	< 0.001	< 0.001	0.693	0.076	**
Treating hospital: 1	< 0.001	0.106	0.096	0.112	*
Treating hospital: 3	< 0.001	0.139	0.139	0.109	*
Treating hospital: 4	0.041	0.001	0.332	0.102	*
**Environmental socioeconomic characteristics**					
Purchasing power per 100 inhabitants	< 0.001	0.719	< 0.001	0.022	*
Unemployment per 100 inhabitants	< 0.001	0.928	0.004	0.014	*

p.adj: *p*-value post-hoc adjusted by Tukey-HSD/ Holm correction for pairwise comparison. * low effect size, ** medium effect size, *** high effect size

### Sociodemographic characteristics

The three groups did not differ significantly regarding gender, children, migration background, school education and professional education. Significant differences were found in the age distribution, marital status, relationship status, living situation, professional situation, degree of employment and the main source of income. However, most effect sizes were small. Cases with conversion of legal status (MHA→LGL cases) were significantly older than cases treated under the MHA or under LGL. This was also reflected in the age group-wise comparison. Over 50% of the MHA→LGL cases were allocated to the age group over 60 years. Meanwhile, MHA→LGL cases were significantly less common in the age group up to 40 years compared to MHA and LGL cases. MHA cases were overrepresented in the middle age group of 41–60 years. LGL cases were more often single and less often married than MHA cases, but cases treated under the MHA lived less often in a relationship compared to MHA→LGL cases. LGL cases lived less often on their own, and MHA cases lived less commonly in assisted accommodation. Regarding the professional situation, MHA cases were more often employed and less often retired compared to LGL and MHA→LGL cases. This was also reflected in the degree of employment. 10.5% of the MHA cases worked full time, while full time employment was practically non-existent among LGL and MHA→LGL cases. Also, over 95% of LGL and MHA→LGL cases reported no employment hours at all compared to 86.7% of MHA cases. This is also reflected in the main source of income which was significantly more often employment for MHA cases and retirement pension for LGL and MHA→LGL cases.

### Clinical and systemic characteristics

No significant differences between the three groups were found for the time of admission, previously attempted suicides, and the reason for detention (danger of self-harm, harm to others, or both). Concerning the main and secondary diagnoses, significant differences were found for the frequency of organic mental disorders including dementia (F0), disorders related to substance use (F1), schizophrenia and other psychotic disorders (F2), and affective disorders (F3). There were no significant differences concerning the rates of comorbidities F1 + F2 and F1 + F6 (personality disorders) between the three groups. F0 diagnoses were most common among MHA→LGL and least common among MHA cases. F1 and F3 diagnoses were most prevalent among MHA cases. Also, F2 diagnoses occurred significantly more often in MHA compared to LGL cases. In regard to other clinical characteristics, LGL cases more often reported previous psychiatric inpatient treatment compared to MHA→LGL cases. LGL cases were also more likely to report previous outpatient treatment than MHA cases. Suicidal tendencies upon admission were more common among MHA compared to LGL cases, but group differences were not significant in the pairwise comparison (Table 3). As with the sociodemographic characteristics, most effect sizes were small.

Regarding systemic characteristics, there were significant differences between the four hospitals. In hospital 1, treatment under the MHA and conversion of legal status (MHA→LGL) were more common with comparably less cases treated under the premises of LGL only. In hospital 3, detention under LGL was more common while in hospital 4, no cases were treated under the premises of LGL at all. Finally, the length of inpatient stay differed significantly with MHA cases staying on average shorter than LGL and MHA→LGL cases. The effect size of the difference of the average length of inpatient stay was moderate (η² = 0.06–0.14).

### Environmental socioeconomic characteristics

No significant differences between the three subgroups were found in the number of businesses, buildings, residential buildings, households, children per 100 inhabitants and the employment rate. LGL cases came from neighbourhoods with higher purchasing power per 100 inhabitants and lower unemployment rates compared to the other two groups.

### Machine learning based case classification

**Fig. 1:**
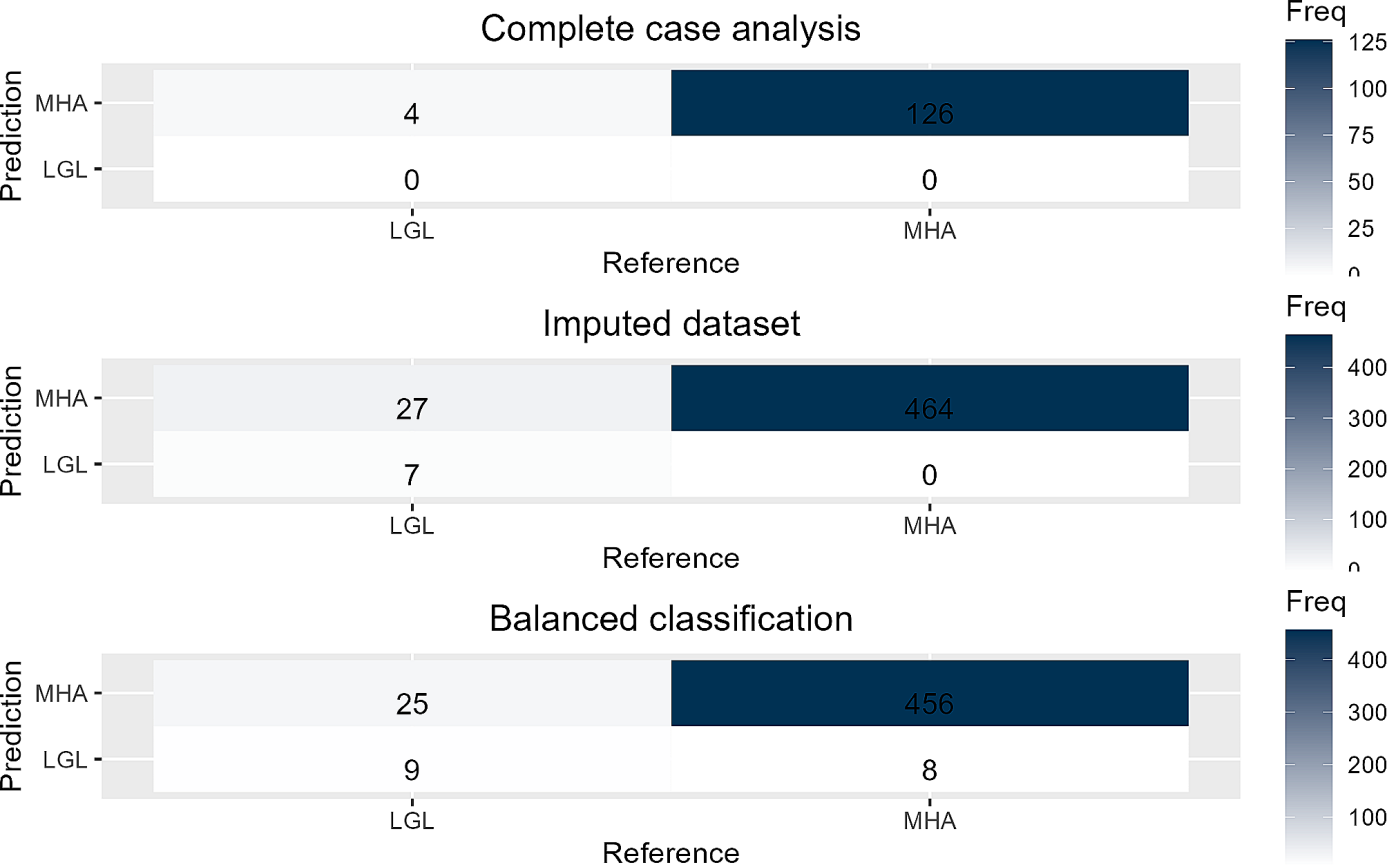
Confusion matrices of the different Random Forest models

The confusion matrices of all three Random Forest models are presented in Fig. 1. All key results of the model performance refer to the algorithm’s application to the test sample. The Random Forest model based on the *complete case dataset* achieved an accuracy of 96.92%, a sensitivity of 0% and a specificity of 100%, which together results in a balanced accuracy of 50%. This is because the Random Forest procedure solely predicted the majority class *MHA*. The area under the curve was 0.573. The Random Forest model on the *imputed dataset* achieved an overall accuracy of 94.58% with a sensitivity of 20.59% and a specificity of 100% resulting in a balanced accuracy of 60.29%. The area under the curve was 0.820. For the *balanced classification dataset*, the imputed training dataset was inflated from 1,164 to 2,168 cases. The SMOTE algorithm thereby achieved an equal distribution of the legal status in the training dataset. After training the Random Forest, it was applied to the same test dataset as with the *imputed dataset* model. The Random Forest model based on the *balanced classification dataset* achieved an overall accuracy of 94.18%, a sensitivity of 29.41% and a specificity of 98.92% leading to a balanced accuracy of 64.17%. The area under the curve was 0.829.

**Fig. 2:**
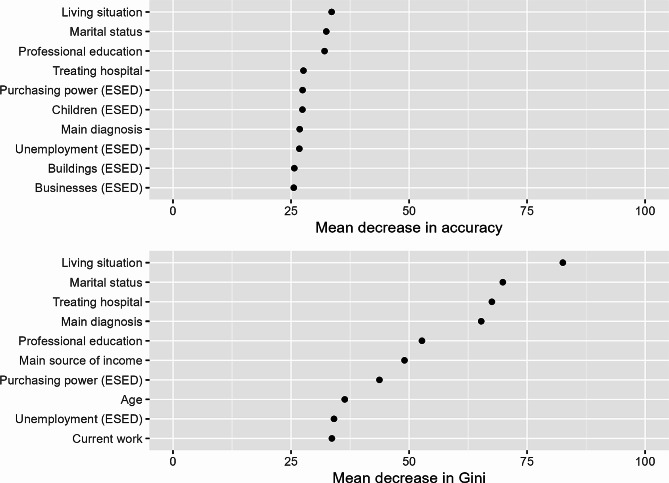
The ten variables with the highest mean decrease in accuracy and mean decrease in Gini of the Random Forest model based on the *balanced classification dataset*

As the Random Forest model based on the *balanced classification dataset* achieved the highest balanced accuracy, we further investigated that model. The ten variables causing the highest mean decrease in accuracy and mean decrease in Gini are shown in Fig. 2. The most relevant sociodemographic, clinical and systemic variables of the model were the living situation followed by the marital status, the treating hospital, the level of professional education, the main diagnosis, the main source of income, the age and the professional situation. The most relevant environmental socioeconomic characteristics of the model were the purchasing power, the number of children per 100 inhabitants, the unemployment rate, and the building and business density per 100 inhabitants.

## Discussion

Despite evidence for the negative impact of involuntary hospitalisation on patients’ trust in the psychiatric system and self-stigma, involuntary hospitalisation remains a commonly used tool to mitigate mental health crises [37, 38]. In the attempt to identify potential preventive measures, there has been substantial research on risk factors for involuntary hospitalisation [14]. However, the majority of research focuses on involuntary hospitalisation under the premises of the MHA although, in an international perspective, other legal frameworks like LGL are applied as well. To our knowledge, the present study is the first study that particularly investigated clinical, systemic, sociodemographic and environmental socioeconomic differences between cases treated under the MHA and LGL. For this, we included all cases treated under the MHA and LGL in the city of Cologne, a densely populated metropolitan area in Germany, in 2011.

Based on our results, a typical MHA patient could be described as a middle-aged male or female with a psychotic, an addiction or an affective disorder. He/ she mostly lives alone or with the family or partner and four out of five patients are unemployed. They often live in districts with a low purchasing power and high unemployment rates, and, on average, they stay half as long in the hospital compared to LGL cases. On the other hand, a typical LGL patient is more often younger and he/ she suffers from a psychotic disorder. They are mostly single and, compared to MHA cases, they more often live in assisted accommodations. They are hardly ever employed and have mostly had previous hospitalisations. Finally, a patient with conversion of legal status from MHA to LGL during the course of hospitalisation is typically an elderly and retired person with an organic mental disorder such as dementia who has not received psychiatric inpatient treatment previously. They usually stay in hospital for about two months, which is longer than the MHA group.

Based on the dataset, we calculated three different machine learning based classification models. The most relevant variables in the model with the highest balanced accuracy were the living situation, the marital status, the treating hospital and professional education. However, the model performance was not satisfactory yielding a balanced accuracy of 64.17% at best.

In conclusion, the three investigated subgroups differed significantly in a number of clinical, sociodemographic and socioeconomic characteristics. Cases treated under the MHA seemed to be less attached to the socio-psychiatric support system. This indicates that studies are warranted to elucidate the reasons for this detachment from services. MHA cases more often suffered from substance use and affective disorders and they commonly expressed suicidal ideations upon admission. This suggests that preventing suicidal ideation in these diagnostic groups may be an effective way to reduce rates of involuntary admissions. In contrast, cases treated under the premises of LGL tended to present with chronic mental health conditions with high proportions of previous psychiatric treatment. This indicates that outpatient and communal services might need to develop additional strategies to avoid readmission in this specific group for example by better tailoring the management of these patients to the individual patient needs. However, one may also argue that readmissions simply cannot be avoided and are probably indicated in this group of severely affected patients. Accordingly, it is possible and plausible that the outpatient and communal services noticed a change in behaviour and initiated the inpatient treatment because of an exacerbation. The chronic nature of the mental health conditions in the LGL group was also reflected by the living situation and the professional situation with a low proportion of cases living on their own, a high number of cases living in assisted accommodation, and low employment rates in this group. Among the LGL cases, psychotic disorders were more common than in MHA cases and they lived in on average less socially disadvantaged districts of the city. This might be caused by the high number of cases in assisted accommodation as these institutions are distributed over all districts. As for the apparent discrepancy of LGL cases being more often retired and hardly ever employed but at the same time living in more wealthy neighbourhoods, we believe that this may be related to the inclusion of cases of comparably wealthy pensioners with organic mental disorders. As we did not have additional information on the respective assets of the patients, but only on the source of income, wealthy individuals that are either retired or do not have a job probably distort these results.

Cases with conversion of the legal status from MHA to LGL in the course of inpatient stay appeared to be a distinctive subgroup. These cases were on average older, and they were most likely to be in a relationship and retired. They also differed significantly from cases who were treated under the premises of LGL since admission in terms of previous treatment, especially inpatient treatment, and they were most likely to suffer from an organic mental disorder. A plausible explanation might be that these cases may represent persons with an initial manifestation of an organic mental disorder, for example a delirium on the basis of dementia or after a stroke, who had not been in touch with the psychiatric system before and therefore did not have a legal guardian. These cases might partially also be referrals from somatic units of other hospitals to bridge the time until the appointment of a legal guardian as a prerequisite for a permanent placement in a retirement home. In practice, this may take several weeks due to the high number of cases pending at the guardianship court. These cases will be difficult to prevent, but improved outpatient services for elder people with organic mental disorders including dementias may be a way to tackle this challenge. This also raises the question, to which extent somatic units specialised on the needs of elderly people with dementia and delirium might help to lower psychiatric admission rates of this patient group, and if better financial compensation of longer inpatient stays of elderly patients in somatic units might also help to lower psychiatric admission rates. Some of these cases probably have extended inpatient stays due to the lack of a suitable retirement home, but the hospitals’ financial compensation is granted based on the medical need for treatment, whereas an insufficient care situation at home does not qualify for inpatient treatment.

The relatively poor model performance of machine learning based models and the mostly low effect sizes of the beforementioned descriptive and inferential analyses imply that the observed differences between the three groups of patients do not entirely explain the different choice of the legal foundation for involuntary psychiatric hospitalisation. However, this is not surprising as we investigated real world data and we should expect a multifactorial aetiology in such a complex issue.

It is however noteworthy that the reason for involuntary treatment (risk of self-harm, harm to others or both) did not differ significantly between the investigated subgroups, although, by German law, LGL is only applicable in cases of risk of self-harm and not in cases of harm to others. This finding is intriguing, especially in view of the fact that the legal basis of a patient treated under LGL because of self-harm cannot be supplemented with a court ruling under the MHA if harm to others becomes immanent during the course of inpatient stay. In fact, as federal law outdoes state law, a court ruling under LGL always unravels an MHA ruling. Together with the significant differences in the distribution of MHA and LGL cases between the four hospitals, this sheds a light on a possibly quite divergent application of the law.

As the German LGL has recently undergone revision [39], the impact of the revised LGL on the clinical practice is yet to be investigated.

### Strengths and limitations

We analysed a comparably large dataset of cases that were involuntarily hospitalised under the MHA and LGL. We were able to include a large variety of clinical, sociodemographic and systemic variables and we enriched the dataset by adding environmental socioeconomic characteristics of the living environment of the patients. To our knowledge, this is the first study focusing on the comparison between MHA and LGL. We were able to minimise distortion by including all cases of an entire year in a metropolitan city as well as distortion due to other systemic factors such as different court rulings. Another strength of our analysis is the application of advanced machine learning based methods such as a Random Forest model, a state-of-the-art machine learning algorithm. We addressed the issue of a high number of missing data which is typical for retrospective analyses by comparing Random Forest models based on a *complete case dataset* with a Random Forest-based *imputed dataset*. We also addressed the imbalanced classification by using SMOTE as it can improve the performance of machine learning algorithms notably when applied to imbalanced healthcare datasets [40, 41].

A main limitation of the study is the questionable generalisability of the results for other regions and countries. In the light of the different handling of LGL cases from hospital to hospital, the situation in other countries with different legal premises might vary even more widely. However, differences in involuntary admission rates between hospitals have been identified in a study from Portugal for MHA cases before, indicating that the hospital may be a modifying factor [42]. Another limitation is that one patient could account for several cases if they were hospitalised multiple times in the study period, and this may have led to an overrepresentation of severe and chronic cases. However, we think that it is important to target these high-users in such analyses, as they are the group which largely determines these processes from the patient side. Furthermore, we were not able to include some potentially important characteristics such as symptom severity and social functioning level based on standardised psychometric instruments. This limitation is related to the retrospective nature of the study, which used clinical routine data that had not been gathered for the primary purpose of research. We are currently collecting data on clinical severity and social functioning level in a continuing quality assurance project in psychiatric hospitals with a view to address this limitation in future studies [43]. The relatively high number of missing values is also related to the use of clinical routine data. We addressed the issue of missing data by means of imputation as dealing with missing data by means of listwise deletion under the Missing at Random (MAR) assumption may cause relevant distortion. However, a possible loss of information or an imputation-based bias of the analysis cannot be ruled out. Finally, as the dataset dates back to 2011, this poses the question whether the results are applicable to today. Since 2011, both the MHA and LGL have undergone revisions. However, the changes concern certain aspects of coercive measures in the course of inpatient stay like the requirement of a separate court order for physical restraints under the MHA and for diagnostics and treatment under coercion in patients hospitalised under LGL. In contrast, legal requirements for the involuntary hospitalisation itself did not undergo relevant revision and the rates for involuntary hospitalisation remained relatively stable [44]. We therefore believe that the results are still valid and do reflect the present psychiatric care situation in Germany.

## Conclusions

In our analysis, cases that were detained involuntarily differed significantly in several characteristics depending on the legal premises of their admission. However, the mostly low effect sizes of the findings and the low balanced accuracies of the Random Forest models indicate that the respective choice of the legal instrument cannot be explained satisfactorily alone with the data available in this study. Hence, systemic differences in respect to the handling of involuntary admissions in different psychiatric hospitals seem to also play a role. In particular, the similarities concerning the reason for admission deserve critical appraisal as involuntary hospitalisation of LCL cases due to danger of harm to others is not stipulated in the German law. We assume that the root of these similarities is both clinically and legally imprecise handling of these cases. Lately, the LGL has undergone revision in Germany, and it now further emphasises that the goal of legal guardianship should be the compliance with the (assumed) wishes of the person under guardianship. Hence, the guardian’s role is to support the people under LG in their own decision making [39]. It deserves further observation to what extent the patients’ characteristics will change in the upcoming years. Furthermore, the application of legal guardianship legislation for the purpose of detention of people with mental disorders appears to vary widely in an international perspective. Therefore, further international research is clearly needed in this field.

## Data Availability

The dataset used for this study contains all involuntarily treated cases of an entire year of four hospitals and includes the number of cases per hospital as well as the respective length of inpatient stay. As it is thereby possible to draw conclusions on the hospitals, the dataset used and analysed during the current study is only available from the corresponding author on reasonable request.

## Abbreviations

ANOVA: Analysis of variance
AUC: Area under the curve
ESED: Environmental socioeconomic data
LGL: German civil code including legal guardianship legislation
MAR: Missing at random
MCAR: Missing completely at random
MHA: Mental Health Act of North Rhine-Westphalia
OOB: Out-of-bag error rate
SMOTE: Synthetic Minority Over-sampling Technique
Tukey-HSD: Tukey’s honest significant differences

## References

[1] Dressing H, Salize HJ (2004). Compulsory admission of mentally ill patients in European Union Member States. Soc Psychiatry Psychiatr Epidemiol.

[2] Sheridan Rains L, Zenina T, Dias MC, Jones R, Jeffreys S, Branthonne-Foster S (2019). Variations in patterns of involuntary hospitalisation and in legal frameworks: an international comparative study. Lancet Psychiatry.

[3] Wickremsinhe MN (2018). Emergency involuntary treatment law for people with mental disorders: a comparative analysis of legislation in LMICs. Int J Law Psychiatry.

[4] Bundesamt für Justiz. Bürgerliches Gesetzbuch (BGB) § 1831 Freiheitsentziehende Unterbringung und freiheitsentziehende Maßnahmen.

[5] Bundesamt für Justiz. Gesetz über das Verfahren in Familiensachen und in den Angelegenheiten der freiwilligen Gerichtsbarkeit (FamFG) § 321 Einholung eines Gutachtens.

[6] Bundesamt für Justiz. Gesetz über das Verfahren in Familiensachen und in den Angelegenheiten der freiwilligen Gerichtsbarkeit (FamFG) § 312 Unterbringungssachen.

[7] Bundesamt für Justiz. Bürgerliches Gesetzbuch (BGB), § 1832 Ärztliche Zwangsmaßnahmen.

[8] Michael L. Perlin. ‘Striking for the Guardians and Protectors of the Mind’: The Convention on the Rights of Persons with Mental Disabilities and the Future of Guardianship Law; 2012.

[9] Putkonen H, Vollm B (2007). Compulsory psychiatric detention and treatment in Finland. Psychiatr bull.

[10] Huber J, Aguirrebarrena G, Ryan CJ (2022). Algorithm for the use of the Guardianship Act, the Mental Health Act and the Public Health Act in emergency departments in New South Wales. Emerg Med Australas.

[11] Tsoh J, Peisah C, Narumoto J, Wongpakaran N, Wongpakaran T, O’Neill N (2015). Comparisons of guardianship laws and surrogate decision-making practices in China, Japan, Thailand and Australia: a review by the Asia Consortium, International Psychogeriatric Association (IPA) capacity taskforce. Int Psychogeriatr.

[12] Morris NP (2020). Detention without data: Public Tracking of Civil commitment. Psychiatr Serv.

[13] Rissmiller DJ, Musser E, Rhoades W, Rissmiller FR, Steer RA (2001). A survey of use of a durable power of attorney to admit geropsychiatric patients. Psychiatr Serv.

[14] Walker S, Mackay E, Barnett P, Sheridan Rains L, Leverton M, Dalton-Locke C (2019). Clinical and social factors associated with increased risk for involuntary psychiatric hospitalisation: a systematic review, meta-analysis, and narrative synthesis. Lancet Psychiatry.

[15] Weich S, McBride O, Twigg L, Duncan C, Keown P, Crepaz-Keay D (2017). Variation in compulsory psychiatric inpatient admission in England: a cross-classified, multilevel analysis. Lancet Psychiatry.

[16] McSwiggan S, Meares S, Porter M (2016). Decision-making capacity evaluation in adult guardianship: a systematic review. Int Psychogeriatr.

[17] Schmitz-Buhl M, Gairing SK, Rietz C, Häussermann P, Zielasek J, Gouzoulis-Mayfrank E (2019). A retrospective analysis of determinants of involuntary psychiatric in-patient treatment. BMC Psychiatry.

[18] Karasch O, Schmitz-Buhl M, Mennicken R, Zielasek J, Gouzoulis-Mayfrank E (2020). Identification of risk factors for involuntary psychiatric hospitalization: using environmental socioeconomic data and methods of machine learning to improve prediction. BMC Psychiatry.

[19] Peters SJ, Schmitz-Buhl M, Karasch O, Zielasek J, Gouzoulis-Mayfrank E (2022). Determinants of compulsory hospitalisation at admission and in the course of inpatient treatment in people with mental disorders-a retrospective analysis of health records of the four psychiatric hospitals of the city of Cologne. BMC Psychiatry.

[20] World Health Organization (WHO). International Statistical Classification of Diseases and Related Health Problems 10th Revision. https://icd.who.int/browse10/2019/en. Accessed 27 Sep 2020.

[21] RWI, Microm. RWI-GEO-GRID: Socio-economic data on grid level- scientific Use file (wave 8). RWI – Leibniz Institute for Economic Research; 2019.

[22] Breidenbach P, Eilers L, RWI-GEO-GRID (2018). Socio-economic data on grid level. Jahrb Natl Stat.

[23] Maremmani AGI, Rugani F, Bacciardi S, Rovai L, Pacini M, Dell’Osso L, Maremmani I (2014). Does dual diagnosis affect violence and moderate/superficial self-harm in heroin addiction at treatment entry?. J Addict Med.

[24] Lee-Winn AE, Mendelson T, Johnson RM (2018). Associations of personality traits with marijuana use in a nationally representative sample of adolescents in the United States. Addict Behav Rep.

[25] Gouzoulis-Mayfrank E (2004). Doppeldiagnose psychose und sucht. Von den Grundlagen Zur Praxis. [Dual diagnosis of psychosis and addiction. From principles to practice]. Nervenarzt.

[26] Fazel S, Långström N, Hjern A, Grann M, Lichtenstein P (2009). Schizophrenia, substance abuse, and violent crime. JAMA.

[27] Bender R, Lange S (2001). Adjusting for multiple testing–when and how?. J Clin Epidemiol.

[28] Breiman L (2001). Random forests. Mach Learn.

[29] Lemon SC, Roy J, Clark MA, Friedmann PD, Rakowski W (2003). Classification and regression tree analysis in public health: methodological review and comparison with logistic regression. Ann Behav Med.

[30] Marchi M, Galli G, Fiore G, Mackinnon A, Mattei G, Starace F, Galeazzi GM (2022). Machine-learning for prescription patterns: Random Forest in the prediction of dose and number of antipsychotics prescribed to people with Schizophrenia. Clin Psychopharmacol Neurosci.

[31] Maito MA, Santamaría-García H, Moguilner S, Possin KL, Godoy ME, Avila-Funes JA (2023). Classification of Alzheimer’s disease and frontotemporal dementia using routine clinical and cognitive measures across multicentric underrepresented samples: a cross sectional observational study. Lancet Reg Health Am.

[32] Danielsen AA, Fenger MHJ, Østergaard SD, Nielbo KL, Mors O (2019). Predicting mechanical restraint of psychiatric inpatients by applying machine learning on electronic health data. Acta Psychiatr Scand.

[33] Wilkinson L (1999). Statistical methods in psychology journals: guidelines and explanations. Am Psychol.

[34] Shah AD, Bartlett JW, Carpenter J, Nicholas O, Hemingway H (2014). Comparison of random forest and parametric imputation models for imputing missing data using MICE: a CALIBER study. Am J Epidemiol.

[35] van Buuren S (2021). Flexible imputation of missing data.

[36] Chawla NV, Bowyer KW, Hall LO, Kegelmeyer WP (2002). SMOTE: synthetic minority over-sampling technique. jair.

[37] Jones N, Gius BK, Shields M, Collings S, Rosen C, Munson M (2021). Investigating the impact of involuntary psychiatric hospitalization on youth and young adult trust and help-seeking in pathways to care. Soc Psychiatry Psychiatr Epidemiol.

[38] Rüsch N, Müller M, Lay B, Corrigan PW, Zahn R, Schönenberger T (2014). Emotional reactions to involuntary psychiatric hospitalization and stigma-related stress among people with mental illness. Eur Arch Psychiatry Clin Neurosci.

[39] Henking T (2022). Die Reform Des Betreuungsrechts. [The reform of guardianship law]. Nervenarzt.

[40] Hassanzadeh R, Farhadian M, Rafieemehr H (2023). Hospital mortality prediction in traumatic injuries patients: comparing different SMOTE-based machine learning algorithms. BMC Med Res Methodol.

[41] Kumar V, Lalotra GS, Sasikala P, Rajput DS, Kaluri R, Lakshmanna K (2022). Addressing Binary classification over Class Imbalanced Clinical datasets using computationally Intelligent techniques. Healthc (Basel).

[42] Silva M, Antunes A, Azeredo-Lopes S, Loureiro A, Saraceno B, Caldas-de-Almeida JM, Cardoso G (2021). Factors associated with involuntary psychiatric hospitalization in Portugal. Int J Ment Health Syst.

[43] Lehmann I, Zielasek J, Blumenröder T, Engemann S, Vrinssen J, Gaebel W (2023). Development and implementation of quality indicators in a group of nine psychiatric hospitals. Z Evid Fortbild Qual Gesundhwes.

[44] Steinert T, Hirsch S, Flammer E (2022). Monitoring Von Zwangsmaßnahmen Und Zwangsbehandlungen in Deutschland. [Monitoring of coercive measures and compulsory treatment in Germany]. Nervenarzt.

